# The propensity of the bacterial rodlin protein RdlB to form amyloid fibrils determines its function in *Streptomyces coelicolor*

**DOI:** 10.1038/srep42867

**Published:** 2017-02-17

**Authors:** Wen Yang, Joost Willemse, Elizabeth B. Sawyer, Fei Lou, Weibin Gong, Hong Zhang, Sally L. Gras, Dennis Claessen, Sarah Perrett

**Affiliations:** 1 National Laboratory of Biomacromolecules, CAS Center for Excellence in Biomacromolecules, Institute of Biophysics, Chinese Academy of Sciences, Beijing, 100101, China; 2 Molecular Biotechnology, Institute of Biology, Leiden University, 2333 BE Leiden, the Netherlands; 3 University of the Chinese Academy of Sciences, Beijing, 100049, China; 4 Department of Chemical and Biomolecular Engineering and Bio21 Molecular Science and Biotechnology Institute, The University of Melbourne, Parkville, 3010 VIC, Australia

## Abstract

*Streptomyces* bacteria form reproductive aerial hyphae that are covered with a pattern of pairwise aligned fibrils called rodlets. The presence of the rodlet layer requires two homologous rodlin proteins, RdlA and RdlB, and the functional amyloid chaplin proteins, ChpA-H. In contrast to the redundancy shared among the eight chaplins, both RdlA and RdlB are indispensable for the establishment of this rodlet structure. By using a comprehensive biophysical approach combined with *in vivo* characterization we found that RdlB, but not RdlA, readily assembles into amyloid fibrils. The marked difference in amyloid propensity between these highly similar proteins could be largely attributed to a difference in amino acid sequence at just three sites. Further, an engineered RdlA protein in which these three key amino acids were replaced with the corresponding residues from RdlB could compensate for loss of RdlB and restore formation of the surface-exposed amyloid layer in bacteria. Our data reveal that RdlB is a new functional amyloid and provide a biophysical basis for the functional differences between the two rodlin proteins. This study enhances our understanding of how rodlin proteins contribute to formation of an outer fibrillar layer during spore morphogenesis in streptomycetes.

A variety of microorganisms can establish a complex, yet highly ordered extracellular matrix in response to various environmental cues. This matrix serves multiple purposes for microorganisms and can alter the surface properties of the enveloped structure^1^, facilitate spore dispersal into the air^2^, mediate cell attachment to surfaces^3^ and in some cases help pathogens to invade their host^4^. The specific composition of this matrix varies from species to species, but these materials often share a highly ordered, fibrous protein component known as amyloid fibrils^5^. Amyloids possess a characteristic cross-β structure, which gives the fibrils both physical strength and chemical stability^6^. Amyloid formation has traditionally been associated with a range of neurodegenerative diseases in humans, yet an increasing number of studies indicate that amyloid proteins often functionally contribute to the physiological well-being of organisms^7^. Within the diverse list of functional amyloids identified over the last decade or so, a significant number have been reported in bacteria, including the well-studied *Escherichia coli* curli fibrils and the TasA amyloids in *Bacillus* biofilms^5^,^8^,^9^. Functional amyloids are also formed by streptomycetes. In *Streptomyces* the aerial mycelium is covered with an amyloidal surface layer, called the rodlet layer, which appears as a network of pair-wise aligned nanorods^10^. Two kinds of secreted proteins, named rodlins and chaplins, are essential for the formation of this rodlet layer. A series of genetic and ultrastructural studies led to a model in which the chaplin proteins serve as the functional amyloid component^11^,^12^,^13^. Chaplins are secreted by growing aerial hyphae to form a tightly-packed fibrillar layer rendering the surface of aerial hyphae and spores hydrophobic, which is required for efficient aerial growth^13^,^14^,^15^,^16^. Rodlins were found to be less crucial for aerial hyphae formation, and were suggested to align chaplin fibrils into the higher order rodlet structure^12^,^17^. Notably, both of the rodlin proteins (RdlA and RdlB) are indispensable for formation of rodlets in the wild-type strain^11^,^16^. Despite their high degree of sequence similarity, the *in vivo* data strongly imply that RdlA and RdlB may each have a unique functional contribution to the structural integrity of the rodlet layer, which cannot be compensated for by the other rodlin protein.

In contrast to the chaplin proteins, the biochemical characterization of rodlins has so far been limited. This is largely due to the fact that the trifluoroacetic acid (TFA) extraction procedure used to isolate rodlins directly from the insoluble cell wall components of *S. coelicolor* results in a mixture of the two rodlin proteins in a completely denatured state^12^. The large size of rodlins compared to the chaplins also makes the proteins less amenable to peptide synthesis. In this study, we have thoroughly investigated the biophysical properties of the RdlA and RdlB proteins expressed recombinantly in *E. coli*. Using a variety of biophysical methods we discovered that RdlB, but not RdlA, readily self-assembles into amyloid fibrils that closely resemble the fibrils observed on the cell surface of aerial structures. The difference in the propensity of the two rodlins to assemble into amyloid fibrils is attributed to small differences in the N-terminal part of the two rodlin proteins. The introduction of three amino acid changes in the N-terminus of RdlA was not only sufficient to impart the ability to form amyloid fibrils *in vitro*, but also restored the formation of the rodlet layer *in vivo* in a strain lacking both RdlA and RdlB. These data show RdlB to be a new functional amyloid, and provide a biophysical basis for the functional differences between the two rodlin proteins.

## Results

### RdlA and RdlB are highly similar

**Figure 1:**
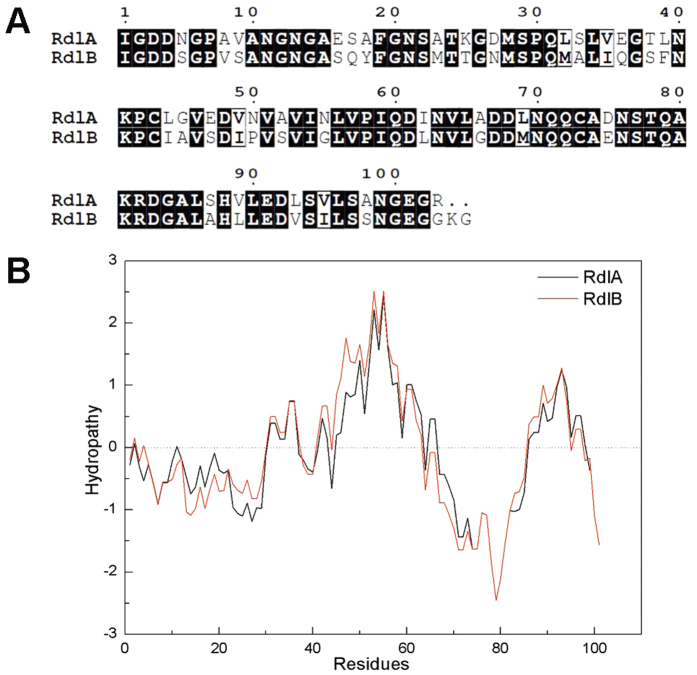
(**A**) The ESPript output of the mature rodlin sequences aligned by ClustalW. Conserved residues have a black background: 71 out of 105 of residues in RdlB are identical to RdlA. (**B**) The hydropathy patterns of RdlA (black) and RdlB (red) appear to be highly similar.

The rodlin proteins RdlA and RdlB of *S. coelicolor* consist of 131 and 133 amino acids, respectively. Lalign analysis^18^ of the mature proteins indicates that the rodlins are 69.6% identical and 93.1% similar (Fig. 1A). The two rodlins also share highly similar hydrophobicity profiles based on the Kyte & Doolittle scale^19^ (Fig. 1B). Notably, RdlA possesses significantly more polar or charged residues near its N-terminus compared to RdlB. As a result, RdlB has a lower net charge and a greater hydrophobic score than RdlA.

TANGO was used to predict the secondary structure of RdlA and RdlB^20^,^21^. TANGO calculation suggests that the regions between residues 51 and 57 in both RdlA (VAVINLV) and RdlB (VSVIGLV) have a high propensity for β-sheet aggregation. The algorithms AGGRESCAN^22^ and FoldAmyloid^23^ which predict aggregation-prone regions showed agreement with the results obtained using TANGO.

### Common features of RdlA and RdlB in solution

**Figure 2:**
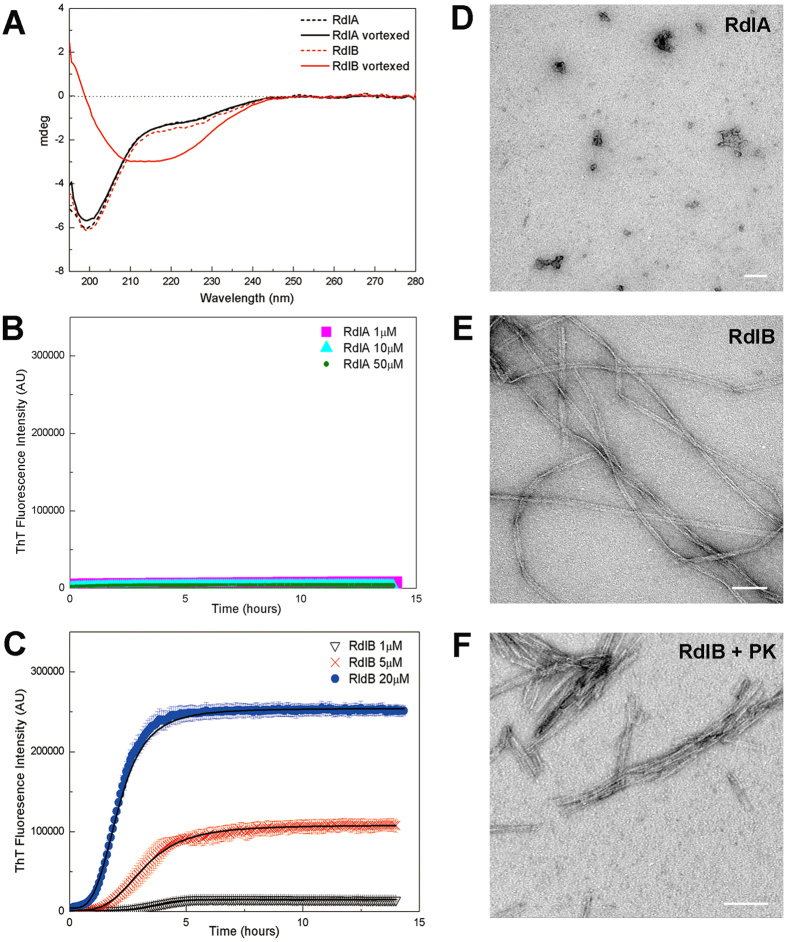
RdlA and RdlB have a different propensity to assemble into amyloid-like fibrils. (**A**) Vortexing induces RdlB to adopt β-sheet rich secondary structure (red solid line) from random coil (red dash line), while RdlA (black solid line) retains the random coil conformation observed before vortexing (black dash line). The protein concentration used was 4 μM in 100 mM sodium phosphate buffer (pH 7.0) measured in a 1 mm cuvette. (**B**,**C**) Increase in ThT fluorescence intensity by RdlA (**B**) and RdlB (**C**) at different concentrations. Readings were taken every 3 min for over 15 hours in a microplate reader while shaking at 700 rpm. The data shown are the average of at least 4 replicates and the error bars represent the standard error of the mean. (**D**) No well-defined structure was found in negatively-stained electron micrographs of RdlA (40 μM) after samples were incubated for 24 hours. (**E**) Negatively stained electron micrograph of mature RdlB fibrils (40 μM) incubated for 24 hours. (**F**) Negatively-stained electron micrograph of RdlB fibrils after 24 hours proteinase K digestion; the thin, needle-like fibrils tend to align into bundles. Scale bars represent 100 nm in images (**D**–**F**).

When rodlins are purified from the cell walls of *Streptomyces,* the trifluoroacetic acid extraction process causes denaturation of the proteins, hampering studies of rodlin structure and function^11^. In order to characterize the rodlin proteins, both RdlA and RdlB were produced recombinantly in *E. coli* without their N-terminal signal peptide. The molecular mass values of the purified rodlins, 10605 Da for RdlA and 10799 Da for RdlB, were confirmed by MALDI-TOF (Fig. S1). Both RdlA and RdlB were shown to be intrinsically disordered by far-UV circular dichroism (CD) (Fig. 2A) and ^1^H-^15^N HSQC NMR spectra (Fig. S2).

We then analysed the surface activity of the two proteins by interfacial tension (IFT) measurements. RdlA and RdlB at a concentration of 100 μg/ml lowered the surface tension by 13 mN/m and 18 mN/m, respectively. In comparison, the chaplin peptide, ChpH was more surface active and was capable of altering the interfacial tension from 72 mN/m to 46 mN/m, in agreement with a previous study^24^. These results indicate that the surfactant properties of the rodlins are less potent than those of the chaplin peptides.

### RdlB forms amyloid-like fibrils *in vitro*

The bioinformatics analysis reveals little difference between RdlA and RdlB, and both rodlins were found to be intrinsically disordered in solution. However, when CD spectra were recorded after 10 min of vortexing, a process known to increase the interfacial surface area and promote mixing that can induce fibril formation, a distinct difference was observed between the two rodlins. RdlB readily adopted β-sheet-rich secondary structure, as indicated by the emergence of a strong negative peak near 220 nm (Fig. 2A). In contrast, the CD spectrum of RdlA barely changed after vortexing.

The amyloid binding dye, Thioflavin T (ThT) was used to monitor fibril formation kinetics. No detectible increase in ThT fluorescence was observed for RdlA indicating little polymerization during prolonged incubation with agitation (Fig. 2B). In contrast, the kinetic curves of the polymerization of RdlB showed a significant increase in ThT fluorescence, suggesting the formation of amyloid-like fibrils (Fig. 2C). Within the range of concentrations tested, the kinetics of RdlB fibril formation was concentration-dependent and a short lag time of ~1 hour was observed. This is consistent with the CD results described above showing a conformational change in RdlB upon vortexing. The lag time is interesting as chaplin peptides assemble rapidly without a detectable lag phase, although both systems are sensitive to agitation.

To assess the morphology of the aggregates formed, transmission electron microscopy (TEM) was used. Unlike samples of RdlA in which only a few irregular aggregates were observed (Fig. 2D), abundant fibrils were observed for RdlB (Fig. 2E). Notably, the RdlB fibrils strongly resemble the fibrillar structures observed after isolation of the rodlet layer with mechanical disruption^10^. The typical fibrils formed by RdlB are highly uniform and of significant length, often exceeding 1 μm. Some very well aligned fibrils could be observed in a parallel pattern. Proteinase K digestion^25^ was used to investigate the stability of the RdlB fibrils. The bundles of thin, needle-like structures observed using TEM showed that the polymerization products of RdlB were proteinase K resistant (Fig. 2F), which implies that they have an amyloidal nature with little globular protein structure displayed on the fibril surface, consistent with structural data obtained by CD. Taken together, these data show that RdlB, but not RdlA, is capable of assembling into amyloid-like fibrils.

### X-ray diffraction demonstrates that RdlB fibrils have a cross-β structure

**Figure 3:**
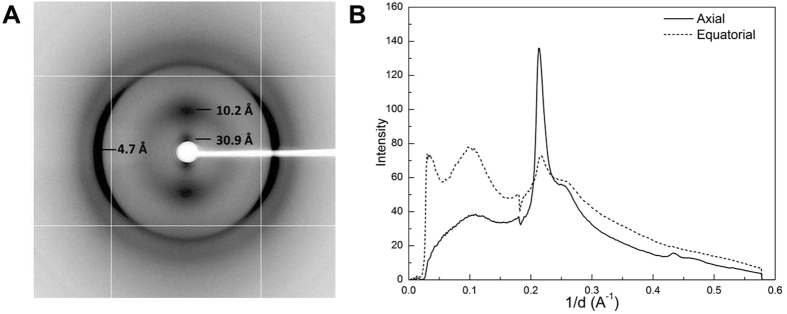
X-ray diffraction pattern of a dried stalk of RdlB fibrils. (**A**) 2-D X-ray diffraction profile with significant reflections marked. (**B**) The reflections in axial and equatorial directions are indicated by the solid and dotted lines, respectively, in the 1-D profile of the X-ray diffraction pattern. The decrease in intensity observed near 5.50 Å (0.18 A^−1^) in both axial and equatorial directions results from the loss of pixels on the image plate shown by the crossed pattern.

The X-ray diffraction pattern of the partially aligned RdlB fibrils displays the features typical of a cross-β structure (Fig. 3). The sharp peak at a spacing of 4.7 ± 0.02 Å indicates the inter-strand distance within β-sheets, while the broad peak centered at 10.2 ± 0.1 Å represents the inter-sheet spacing within the fibril cross-section^26^, confirming that the fibrils formed by RdlB are amyloid fibrils. The reflection at 30.9 ± 1.4 Å in the equatorial axis may arise from the arrangement of multiple protofilaments within the RdlB fibrils^27^. This reflection could also indicate the length of a single β-strand consisting of residues involved in the fibril core^28^. Given that the average distance between residues within a polypeptide chain is 3.8 Å^29^, this observation suggests there might be a 9–10 residue β-aggregation-prone region in the RdlB sequence which directly constitutes the fibril core. It is plausible that the β-strand incorporates only part of the RdlB sequence, with some residues extending from the fibril core remaining disordered and flexible to a certain degree. Fibril stalks formed by chaplins also gave an axial inter-strand reflection at ~4.7 Å and the equatorial inter-sheet reflection at ~10 Å^24^, similar to the X-ray diffraction pattern of RdlB fibrils, but lack equatorial reflections at 30.9 Å, suggesting that the alignment of RdlB fibrils is more ordered than in Chp fibrils.

### RdlB has an amyloid-forming region near the N-terminus

**Figure 4:**
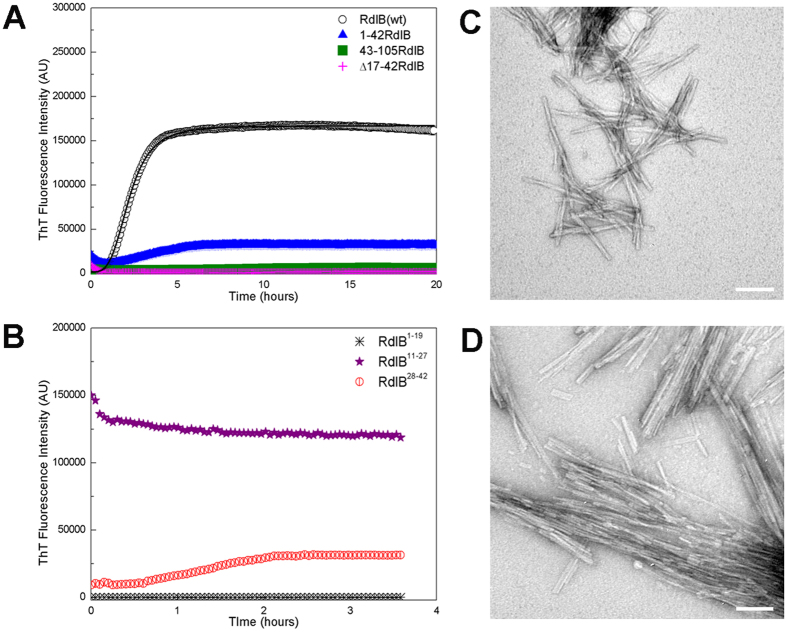
The *in vitro* characterization of RdlB mutant aggregation. (**A**) Aggregation of RdlB and its mutants monitored by ThT fluorescence intensity. All conditions were identical and all protein concentrations 10 μM. (**B**) *In vitro* self-polymerization of three synthetic peptides based on the N-terminal sequence of RdlB monitored by ThT fluorescence. The peptide concentration was 40 μM. (**A**,**B**) Readings were taken every 3 min in a FLUOstar Omega microplate reader with 700 rpm orbital shaking. (**C**,**D**) Negatively stained electron micrograph of aggregates formed by RdlB peptide fragments after incubation for 4 days. Scale bars are 100 nm. (**C**) Peptide corresponding to residues 11–27 of RdlB (NGNGASQYFGNSMTTGN). Fibrils appear as short, rigid rods. (**D**) Peptide corresponding to residues 28–42 of RdlB (MSPQMALIQGSFNKP). Fibrils appear significantly shorter and thinner, the morphology of these fibrils appears more fragile as shreds of fibrils were often observed indicating fragmentation.

Given the high similarity between the sequences of the two rodlin proteins, the difference in aggregation properties of these two proteins was surprising. The N-terminal 50 or so residues is the region of greatest difference between RdlA and RdlB (Fig. 1A), and residues 51–57 in both proteins was predicted to be prone to aggregation. Taking this into account, we first constructed a series of complementary and overlapping fragments of RdlB to narrow down the region(s) of RdlB responsible for amyloid assembly. We found that the N-terminal fragments residues 1–42 (Fig. 4A) and residues 1–58 (not shown) readily formed aggregates, whereas the complementary C-terminal fragments residues 43–105 (Fig. 4A) and residues 59–105 (not shown) did not, narrowing the amyloid fibril forming region to the N-terminal 42 residues. We then further screened truncations in RdlB by deleting regions located near the N-terminus and tested the aggregation propensity of the resulting proteins. Whilst ThT binding can vary between fibrils, this assay together with EM observation provides a good assessment of whether aggregation has occurred. We found that deleting the region between residues 17 and 42 (Δ17-42RdlB) prevented RdlB from assembling into amyloid fibrils. There was little detectable increase in fluorescence of the dye ThT, even after prolonged incubation of Δ17-42RdlB (Fig. 4A). In contrast, two mutants with smaller deletions within this same region, Δ17-29RdlB and Δ31-42RdlB, were still able to assemble into amyloid fibrils (Fig. S3). The mutant Δ51–57RdlB also formed fibrils (Fig. S3), confirming that residues 51–52, which are predicted to be aggregation prone, do not in fact contribute to aggregation of RdlB. Taken together, these results identify the region between residues 17–42 as critical for initiating the polymerization of RdlB.

**Figure 5:**
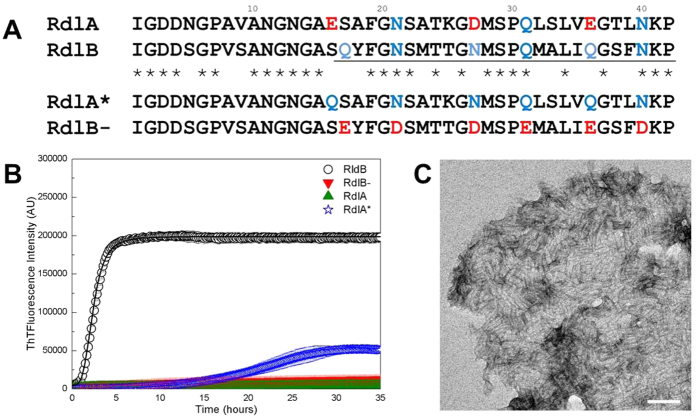
Mutants of RdlA and RdlB exhibited reversed propensity for amyloid formation. (**A**) Sequence alignment of the N-terminal regions of rodlins and the designed mutants. (**B**) The aggregation of wild-type rodlins and the mutants RdlB- and RdlA* monitored by ThT fluorescence over 30 h. The protein concentration for each protein was 10 μM. Readings were taken every 3 min in a FLUOstar Omega microplate reader with 700 rpm orbital shaking. (**C**) Negatively stained electron micrograph of RdlA* (80 μM) after incubation for 6 days with gentle shaking. The scale bar is 100 nm in length.

To further dissect the region important for amyloid fibril formation, short synthetic peptides based on the sequence of the N-terminal region of RdlB were screened for their aggregation capability. The peptide RdlB^11^,^12^,^13^,^14^,^15^,^16^,^17^,^18^,^19^,^20^,^21^,^22^,^23^,^24^,^25^,^26^,^27^ (NGNGASQYFGNSMTTGN) and the peptide RdlB^28^,^29^,^30^,^31^,^32^,^33^,^34^,^35^,^36^,^37^,^38^,^39^,^40^,^41^,^42^ (MSPQMALIQGSFNKP) both showed a significant propensity for amyloid aggregation in a ThT fluorescence assay (Fig. 4B). The RdlB^11^,^12^,^13^,^14^,^15^,^16^,^17^,^18^,^19^,^20^,^21^,^22^,^23^,^24^,^25^,^26^,^27^ fibrils observed by TEM appeared to associate into tangled networks with some fibrils displaying a slight twist (Fig. 4C). Meanwhile, fibrils formed by the peptide RdlB^28^,^29^,^30^,^31^,^32^,^33^,^34^,^35^,^36^,^37^,^38^,^39^,^40^,^41^,^42^, displayed a greater tendency to align in parallel clumps (Fig. 4D). The ThT results and EM examination suggest that the two peptides both contain residues that directly contribute to the amyloid formation propensity in full length RdlB, although the properties of the resulting fibrils differ. In contrast, the first 19 residues of RdlB (IGDDSGPVSANGNGASQYF) was not able to assemble into amyloid fibrils suggesting these residues are less important to fibril formation (Fig. 4B). Therefore, a 26 residue amyloid determining region was defined as residues 17–42 in the N-terminal of RdlB (Fig. 5A).

### The propensity to form amyloid fibrils can be swapped between RdlA and RdlB

We then compared the sequences of the two rodlins within this 26 residue amyloid region at the residue level. Although the overall hydropathy plots of the N-terminal regions of the two rodlins are similar (Fig. 1B), RdlA possesses more charged residues compared to RdlB (Fig. 5A). In particular, RdlA has glutamate or aspartate residues at positions 16, 27 and 36, and these long charged side chains are generally considered to disfavor β-sheet stacking^20^,^30^. In RdlB, the residues at the corresponding sites are serine, glutamine or asparagine, which are considered more favourable for β-sheet formation^31^. We therefore constructed a RdlA mutant, named RdlA*, which contains the three site mutations E16Q, D27N and E36Q, corresponding to the differences between the wild-type RdlA and RdlB sequences. In parallel, we created the corresponding RdlB variant protein, RdlB*, in which the three putative amyloid-favoring residues were replaced by glutamate or aspartate residues, as found in the RdlA sequence. However, as these three changes in RdlB* did not completely remove amyloid forming ability *in vitro* (Fig. S4), we introduced an additional three charged residues into RdlB*, yielding RdlB− (Fig. 5).

The amyloid propensity was strongly diminished in RdlB−, and was comparable to that of RdlA, suggesting there is little accumulation of amyloid-like structure (Fig. 5B). Conversely, the RdlA* mutant gained the propensity to form amyloid-like structures, as deduced from the significant increase in ThT fluorescence. Even though the self-assembly of RdlA* revealed an extended lag time and a longer exponential phase compared to RdlB, TEM analysis confirmed the formation and presence of abundant fibrillar structures (Fig. 5C); the observed fibrils of RdlA* were relatively short and tended to clump together to form a large tangled structure, possibly reflecting the relatively high protein concentration used to obtain sufficient material for EM analysis. Taken together, these data highlight these three residues as crucial determinants for the different propensity of RdlA and RdlB to assemble into amyloid fibrils, namely residues at positions 16, 27 and 36.

### The amyloid propensity of rodlins correlates with the presence of rodlets

**Figure 6:**
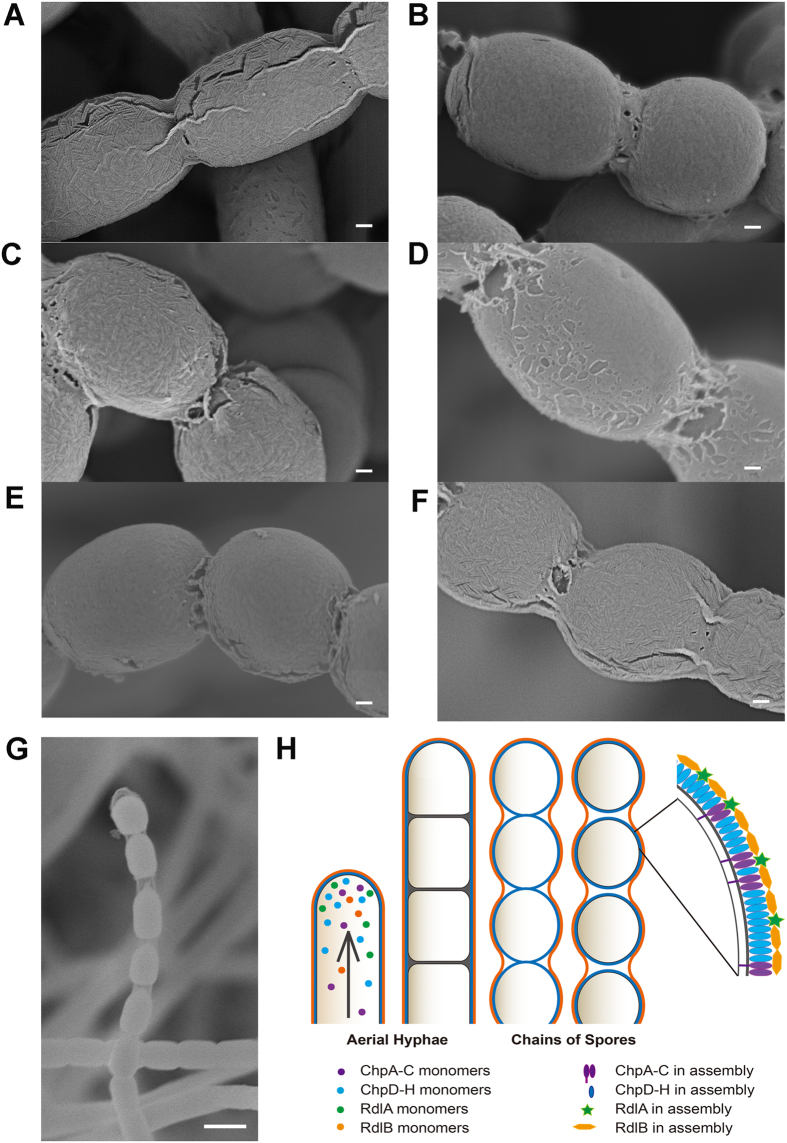
The propensity of rodlins to form amyloid fibrils correlates with the presence of rodlets. The outer surface of spores of the wild-type strain (**A**) is characterized by the rodlet layer. In contrast, no rodlets are detected on the surface of spores of the ∆*rdlB* strain (**B**). Introduction of the wild-type *rdlB* gene (contained on plasmid pIJ8630-rdlB) in the ∆*rdlB* strain restored rodlet formation (**C**). No complementation is observed when pIJ8630-∆17-42rdlB (**D**) or pIJ8630-rdlB− (**E**) are introduced in the *∆rdlB* mutant strain. (**F**) Rodlets are also formed by the introduction of plasmid pIJ8630-rdlA*2 in the ∆*rdlAB* mutant. (**G**) Spore chains of the *S. coelicolor* wild-type strain as observed with scanning electron microscopy without platinum plasma coating. A transparent sheath-like structure is visible surrounding the separating spores. (**H**) Proposed model for the developmental transition of *S. coelicolor* aerial hyphae into chains of spores, which are enveloped by two amyloidal layers containing assembled chaplins (blue/purple) and rodlins (orange/green). Note that the rodlins are part of the outermost layer. The scale bar represents 100 nm (**A**–**F**) or 1 μm (**G**).

Previous work indicates that both RdlA and RdlB are essential for rodlet assembly. Given that RdlB exhibits strong fibril forming capability *in vitro*, we created *Streptomyces* strains that express variants of RdlA or RdlB with altered amyloid-forming propensity. The rodlet layer, which is described as a sheet of ordered rods in a mosaic pattern, is typically observed on the outer surface of aerial mycelia and spore chains in *S. coelicolor* (Fig. 6A). In the ∆*rdlB* strain, the surface of the spores appeared smooth without a detectable fibrous structure, even though it is suggested to be covered with chaplin proteins in a fibrillar form (Fig. 6B). In agreement with earlier work, we first confirmed that the rodlet layer was restored in the ∆*rdlB* strain after reintroduction of the wild-type *rdlB* gene^12^ (Fig. 6C). In contrast, the introduction to the ∆*rdlB* strain of plasmid pIJ8630-∆17-42rdlB, which expresses the RdlB mutant lacking amyloid fibril forming capability, failed to restore the formation of rodlets *in vivo* (Fig. 6D). This confirms that this 26 residue amyloid region in RdlB is crucial for formation of the rodlet layer. Also, no rodlets were detected on the spore surface when the *∆rdlB* strain was complemented with pIJ8630-rdlB*−*, which leads to the production of the RdlB− mutant protein with a diminished propensity to form amyloid fibrils (Fig. 6E). The secretion and correct localization of the RdlB− mutant along with RdlA were confirmed by MALDI-TOF mass spectrometry of spores collected from the complemented strain (Fig. S5A)^32^.

To see if the amyloid-promoting mutations in RdlA* were also functional *in vivo*, we introduced rdlA* in the *∆rdlAB* strain that lacks the rodlet pattern (Fig. S6A). Given our previous observation that the absence of either rodlin gene affects the expression level of the remaining copy^12^, two copies of rdlA* were introduced to ensure sufficient production of the mutant RdlA* protein to potentially compensate for both RdlA and RdlB, and intriguingly, rodlets were evident on the spore surface of the complemented mutant (Fig. 6F). The rodlet pattern, in terms of both the coverage and the paired alignment of the fibrous structure, observed on the RdlA* expressing strain (Fig. 6F) closely resembled the rodlet layer of the wild-type strain (Fig. 6A), or the *∆rdlAB* mutant complemented with the pIJ8630-rdlArdlB plasmid that restores both RdlA and RdlB (Fig. S6B). MALDI-TOF mass spectrometry of spores of the complemented strain indicated that RdlA* was correctly localized on the spore surface with RdlB absent in this strain (Fig. S5B). Taken together, these data show that the ability of RdlB or RdlA* to form amyloid fibrils plays an instrumental role in rodlet formation on the cell surface. Further, the rodlins may contribute to formation of the thin sheath-like structure that has been previously reported to encapsulate the spores^39^,^40^, and which we also detected using scanning electron microscopy (SEM) imaging without applying the typical plasma coating (Fig. 6G).

## Discussion

Functional amyloids have been recognized in a wide range of prokaryotic and eukaryotic organisms. In streptomycetes, functional amyloids formed by the chaplin proteins confer surface hydrophobicity to aerial structures, which may facilitate spore dispersal. Here, we demonstrate that the rodlin protein RdlB, also assembles into amyloid fibrils, while RdlA shows no fibril-forming capability. The difference in amyloid propensity of the rodlin proteins arises from a few amino acid differences within the N-terminal regions of these two proteins. Notably, three amino acid changes were sufficient to change the non-assembling RdlA into a functional amyloid. Our data provide new insight into the regulation of amyloid assembly, and the organization of the surface layers enveloping aerial hyphae in streptomycetes.

### RdlB forms functional amyloid fibrils

We purified rodlin proteins produced recombinantly in *E. coli*, and found them to be natively disordered. Such intrinsically disordered proteins have significant structural plasticity, allowing them to adopt different tertiary structures, depending on the environment and specific ligands available for binding^33^. RdlB consists primarily of random-coil structure in its native soluble state, similar to many other amyloidogenic polypeptides, yet it can readily form β-sheet structure and polymerize into amyloid fibrils. Amyloid aggregation is often initiated by a relatively small region of the polypeptide chain, referred to as an aggregation nucleus^21^. For RdlB, a 26 residue region located in the N-terminus was identified as the crucial amyloid-forming region (residues 17–42). Interestingly, this region was not detected by the β-sheet or amyloid formation propensity prediction algorithms TANGO, AGGRESCAN or FoldAmyloid, which identified only a 7 residue region (VSVIGLV) corresponding to residues 51–57 of RdlB as highly prone to aggregation. Those algorithms tend to give weight to hydrophobic residues when locating aggregation-prone clusters in protein sequences so this difference is not unexpected nor without precedent. The 7 residue region identified by the algorithms was initially of interest because it contains the motif SVIGL, which is also present in the amyloid-determining region of ChpH^34^. However, without these residues RdlB (∆51-57RdlB) was still able to form fibrils efficiently (Fig. S3) and the C-terminal fragment of RdlB (i.e. 43-105RdlB) is incapable of forming fibrils. Further, the same region, with similar sequence, is also predicted to be aggregation prone in RdlA, but RdlA is unable to form fibrils. Thus we conclude that the aggregation-prone VSVIGLV sequence does not provide the crucial driving force for RdlB amyloid formation, although this region may favor the association of RdlB monomers by hydrophobic stacking.

Two sub-regions within the 26 residue amyloidogenic region identified in RdlB, residues 17–29 (QYFGNSMTTGNMS) and residues 31–42 (QMALIQGSFNKP), were individually able to promote the fibril formation of the RdlB protein (Fig. S3). We propose that these two sub-regions can individually form β-strands and together adopt a β-hairpin structure, serving as the structural template for polymerization of full length RdlB. In comparison with RdlA, the lower density of charged residues was the main distinguishing feature of this region. This is supported by the observation that the RdlB variant RdlB−, in which six polar residues in this region were replaced by charged residues, no longer assembles into amyloid fibrils *in vitro* and *in vivo*. Conversely, the ability to form amyloid fibrils is conferred to RdlA*, a variant of RdlA in which three charged residues that strongly disfavor amyloid aggregation were replaced with the hydrogen bond favoring residues present in RdlB. Interestingly, with its newly acquired amyloid formation propensity, RdlA* was able to rescue the rodlet morphology in the ∆*rdlAB* mutant (Fig. 6F), which wild-type RdlA is unable to do^12^. Taken together, we have demonstrated a correlation between amyloid-forming propensity and the presence of a fibrous rodlet structure on mature spores, and identify RdlB as a functional amyloid involved in extracellular coating in *S. coelicolor.*

### The role of RdlA in rodlet layer assembly remains elusive

Our *in vitro and in vivo* data clearly demonstrate that RdlB forms amyloid fibrils that are required for formation of the rodlet layer in the wild-type bacterium. However, the *in vivo* function of RdlB also requires RdlA. This is concluded from the fact that the rodlet layer is absent in the *∆rdlA* strain, whether or not it is supplemented with an additional copy of the *rdlB* gene^12^. In other words, with a high amyloid-forming propensity, the protein RdlB itself cannot form the rodlet layer. Given that RdlA and RdlB are both abundantly present on the cell surface^11^, and that only RdlB appears to form amyloid fibrils, the question remains: how does RdlA contribute to rodlet formation? One classic example of the coordination between homologous proteins in extracellular functional amyloid assembly is the *E. coli* curli system, where CsgB functions as a nucleator for CsgA^35^,^36^. However, such cooperation does not seem to apply to RdlA and RdlB, as no seeding effect was observed when RdlB fibril seeds were added into a RdlA protein solution (Fig. S7A). Furthermore, the presence of RdlA did not interfere with the tendency of RdlB to form amyloid fibrils (Fig. S7B). However, due to the unknown dynamic stoichiometry of the two homologous rodlin proteins during aerial growth, it is hard to exclude the possibility that RdlA may be indispensable for achieving the appropriate assembly speed of RdlB fibrils. It is plausible that RdlA serves as a “regulator” *in vivo* by interfering within the expression pathway to avoid the possible cytotoxicity or incorrect localization of amyloid oligomers due to the premature self-aggregation of RdlB, as an autoregulatory system is necessary for precise functional protein assembly^37^. However, titration of RdlA and RdlB had no effect on the position of the NMR peaks (data not shown), which indicates that the two proteins do not interact directly in solution. We therefore assume that the coordination between the two rodlins *in vivo* may require participation of additional components present in the extracellular matrix. Indeed, as a smooth sheath-like outer layer could be observed for the strain lacking rodlins, it is suggested that other yet unidentified components contribute to the rodlet layer in addition to rodlins in their fibrillar form (see below).

### Developing aerial structures are enveloped by two amyloid layers

Aerial growth in *S. coelicolor* is initiated by the accumulation of sufficient amounts of chaplins, in particular the small chaplins ChpE and ChpH, at the water-air interface, which contributes to lowering of the surface tension. The surfactant molecule SapB has also been implicated in this process, although its function appears to be dependent on the growth medium^15^,^32^. The rodlins appear to function at a later developmental stage. This is based on the fact that i) the expression of the rodlin genes is induced after the hyphae have left the aqueous environment^12^,^38^, and ii) the surface activity of rodlins is not as great as that of the chaplins or SapB. The later involvement of rodlins in spore morphogenesis is consistent with the finding that the formation of aerial hyphae is unaffected in mutant strains lacking either or both rodlin genes under normal conditions^12^. Following secretion, rodlins are found localized on the outer surface as abundant components of the protein fraction of aerial hyphae that is SDS-insoluble, but extractable with TFA^11^. Notably, in the absence of the rodlin proteins, rodlets that normally decorate the outer surface of aerial hyphae and spores are no longer formed; rodlets are also absent when the chaplin genes were inactivated, which lead to a model in which both proteins cooperatively form the rodlet layer^12^. However, the work presented here favors a model in which the rodlet layer may in fact be a separate layer on top of the amyloidal layer first formed by the chaplin proteins (Fig. 6H). This is supported by the observation that spores appear to be encapsulated by a thin sheath-like structure^39^,^40^ (Fig. 6G). Secondly, RdlB and also RdlA* assemble both *in vivo* and *in vitro* into pair-wise fibrils that are very similar in appearance to the rodlets on the wild-type cell surface. Thirdly, the presence of two layers is supported by previous assessments of β-sheet structure *in situ* on the spore surface by FTIR microscopy^24^. β-Sheet structure was observed in the wild-type strain and in the ∆*rdlAB* mutant where rodlins are lacking but not in ∆*chpABCDEFGH* mutant lacking the chaplins^24^. This implies that the assembly of rodlins *in vivo* depends on the chaplins. Taken together, these arguments favor a model where two surface layers envelope developing aerial hyphae (Fig. 6H).

What would be the advantage of having two amyloid layers enveloping aerial hyphae? We propose that the amyloid layer formed by the chaplin proteins provides both surface hydrophobicity to the spores, as well as protection. The rodlin layer, on the other hand, may provide an extra layer that ensures that developing spores remain associated with the colony and are not released before the sporulation process has been completed. Sporulation leads to the synchronous formation of a hundred or so division sites in the aerial hyphae, a process that is coordinated by the SsgA-like proteins^41^. Eventually, these spores will become separated from each other and distributed to other sites to establish new colonies. It is important that only properly matured spores leave the colony. The rodlins may contribute to the strength and hydrophobicity of an additional surface layer that only ruptures when the sporulation process is complete. Indeed, complete spore chains were found to be surrounded by a layer that appeared distinct from the layer that envelopes the individual spores^10^. This hypothesis would also explain why ruptures in the rodlet layer are often evident with scanning EM, in particular at the sites connecting two adjacent spores. Such ruptures would not compromise the mature spores as they still have an intact amyloid layer formed by the underlying chaplin coating. Additionally, this would ensure that the surface hydrophobicity of spores is maintained in the ∆*rdlAB* mutant, even though the spores lack their rodlet pattern^11^.

The biophysical characterization of the rodlin proteins from *S. coelicolor* reveals an unexpected divergence in the properties of the two highly homologous proteins. As we have shown here for the first time, RdlB forms amyloid fibrils that are essential for the structural integrity of the rodlet layer in *S. coelicolor*. This represents a significant development in terms of guiding further investigation of the assembly of the rodlet layer in *Streptomyces*. Besides the identification of a new functional amyloid, our results also indicate that *S. coelicolor*, as it recruits multiple amyloids for its extracellular matrix assembly, could be an important model for further study of how functional amyloids are exploited in organisms under intricate regulatory control. In addition, our results are also of importance in understanding the mechanism of amyloid fibril aggregation at the residue level and could provide valuable insight into the differences between pathogenic and functional amyloids.

## Materials and Methods

### Strains and growth conditions

*Streptomyces coelicolor* strains used in this work are listed in Table S1. All *S. coelicolor strains* were cultured at 30 °C on solid MS agar medium^42^. *E. coli* DH5α^43^ was used for plasmid construction, while *E. coli* ET12567/pUZ8002^42^ was used for conjugation as described^42^.

### Plasmids

Plasmids used for the *in vivo* complementation are listed in Table S1. All oligonucleotides used for constructing mutational variants of rodlins listing in Table S2 were obtained from Sangon Biotechnology (Shanghai, China). The mutants of *rdlB* were constructed by site-directed mutagenesis of an EcoRV-flanked 1.4 kb fragment cloned in pBluescript II KS+, encompassing the putative promoter region, the coding sequencing of wild-type *rdlB* and a 748 bp sequence 3′ of the stop codon of *rdlB*^12^. The rdlA* mutant was acquired through site-directed mutagenesis of a 1.5 kb EcoRV-flanked fragment in pBluescript II KS+, containing the putative promoter and coding sequence of *rdlA*, as well as 843 bp sequence 3′ of the stop codon of *rdlA*. The sequences of all mutations were confirmed by bi-directional sequencing. In all plasmids containing the mutant *rdlA* and *rdlB* genes, a 2.2 kb BamHI fragment encompassing the hygromycin resistance gene from plasmid pHP45 Ωhyg^44^ was inserted. The resulting plasmids were cut with KpnI/XbaI to excise fragments carrying the mutant *rdlA* or *rdlB* genes together with the hygromycin resistance gene, and which were subsequently ligated into pIJ8630 cut with the same enzymes^42^.

### Bioinformatics

All input for bioinformatics analysis was according to the previously published sequences of the mature rodlin proteins (accession numbers: RdlA (AJ315950) and RdlB (AJ315951)). The rodlin sequences were characterized without the N-terminal signal peptide (residues 1–28) based on the reported cleavage sites^11^. The Lalign server was employed to produce sequence alignments and identity calculations of rodlins with the default scoring matrix in which the open gap penalty and the extended gap penalty were −14 and −4, respectively^18^. The hydrophobicity scores for rodlins were calculated by ProtScale^45^ based on the Kyte and Doolittle scale^19^ *via* the ExPASy proteomics server. TANGO was used for predicting the secondary structure and the β aggregation propensity^20^,^21^. The parameters for aggregation propensity calculation were 298 K, pH 7.0 and an ionic strength of 0.225 M (which is the ionic strength of a 100 mM sodium phosphate buffer at pH 7.0). The aggregation-prone regions were also predicted using the programs AGGRESCAN^22^ and FoldAmyloid^23^.

### Protein Preparation

Rodlins and mutant variants thereof were recombinantly expressed in *E. coli* without their 28 residue signal sequence. The *rdlA* and *rdlB* genes were obtained by PCR from genomic DNA of *Streptomyces coelicolor* and confirmed by DNA sequencing. Mutants of RdlB were generated using the primers listed in Table S2. Rodlins and mutational variants were cloned into the pET-28a expression vector. The recombinant rodlins containing a His-tagged SMT3 protein followed by a ULP1 cleavage site at the N-terminus. Note that the recombinant rodlins have an extra serine residue at the N-terminus after cleavage of the fusion tag. Proteins were expressed in *E. coli* BL21 (DE3) cultured at 37 °C in 2YT medium supplemented with 200 μg/ml kanamycin till the OD600 of the medium reached 0.7. Protein production was induced with 0.7 mM isopropyl-β-D-thiogalactopyranoside (IPTG) at 18 °C for 12 hours. Cells were harvested at 4800 × *g* at 4 °C for 30 min by centrifugation. Cell pellets were resuspended in 100 mM sodium phosphate buffer, pH 7.0. After cell lysis and centrifugation, the lysate was purified by Ni-column (Chelating Sepharose Fast Flow). Fractions containing the target proteins underwent ULP1 digestion followed by a second Ni^2+^-affinity purification. After the SMT3 protein was removed, the rodlins were concentrated and loaded onto the Superdex G75 column (GE Healthcare) and purified by gel filtration chromatography, except truncated mutant 1-42RdlB which precipitated during the ULP1 digestion due to its strong tendency to rapidly form from fibrils. The buffer for gel filtration and final protein storage was 100 mM sodium phosphate buffer, pH 7.0. The purity of proteins was checked by SDS-PAGE, and the concentrations were determined by BCA assay^46^. Proteins were stored at −80 °C in sodium phosphate buffer directly after flash-freezing.

### Peptide Preparation

Synthetic peptides corresponding to the N-terminus sequence of RdlB were purchased from Sangon Biotechnology (Shanghai, China). The purity (>95%) and composition of the peptides was confirmed by high performance liquid chromatography along with electrospray mass spectrometry.

### Interfacial Tension

The surface activity of both RdlA and RdlB were anlysed by pendant droplet analysis on a contact angle system (Contact Angle System OCA, DataphysicsCo., Germany). The rodlin solution was prepared in 10 mM sodium phosphate buffer pH 7.0 at a concentration of 100 μg/ml. As a control, ChpH was dissolved in the same buffer at the same concentration as RdlA and RdlB. For all measurements, the volumes of the droplets were between 18 μl and 21 μl. All measurements were performed at ambient temperature.

### NMR Spectroscopy

NMR experiments were performed at 298 K on an Agilent DD2 600 MHz NMR spectrometer. NMR samples contained 400 μM ^15^N- labeled rodlin protein in 100 mM sodium phosphate buffer pH 7.0 with 5 mM CHAPS, 4 mM DL-Dithiothreitol, 0.5 mM EDTA and 10% (v/v) D_2_O. All data were processed with NMRPipe^47^ and analyzed with NMRViewJ^48^.

### Circular Dichroism Spectroscopy

Far-UV CD spectra of rodlin solutions were acquired on a Chirascan plus CD spectrometer (Applied Photophysics Ltd., UK). Rodlins were diluted with 100 mM sodium phosphate buffer pH 7.0 to give a final concentration of 4 μM. The spectra were recorded between 195 nm and 280 nm at 25 °C in a 1 mm path length cuvette. The scan rate was 20 nm/min with steps of 0.5 nm. The spectra were generated based on 3 scans per sample and corrected by subtracting the reference spectrum of the sodium phosphate buffer. To determine the secondary structure changes of RdlA and RdlB induced by contact with the water-air interface, agitation was introduced by vortexing vigorously for 10 min. After vortexing, samples were immediately analysed as described above.

### Amyloid Fibril Formation

The kinetics of amyloid fibril formation of RdlA, RdlB and the mutant variants thereof were monitored using Thioflavin T (Sigma-Aldrich)^49^. A 150 μl protein sample in 100 mM sodium phosphate buffer pH 7.0 was mixed with ThT (20 μM) in 96 well plates with an optical flat bottom (Coning, NY, USA) which were subsequently covered with PlateMax film (Coning, NY, USA) to prevent evaporation. The fluorescence was measured using a microplate reader (FLUOstar Omega, BMG, Germany) at 25 °C, with orbital shaking at 700 rpm between readings. Samples were excited at 450 nm, after which the emission was measured at 480 nm. For each sample at least 4 replicates were measured.

### Proteinase K Digestion

Proteinase K (Ameresco) was added to RdlB fibrils (80 μM) after 2 days incubation in 100 mM sodium phosphate buffer pH 7.0 to reach a working concentration of 0.5 mg/ml. CaCl_2_ was added to give a final concentration of 10 mM. Digestion was carried out at room temperature overnight. Samples were then quickly frozen using liquid nitrogen and stored at −80 °C until further use.

### Transmission Electron Microscopy

For TEM examination, samples of RdlA, RdlB and mutants thereof were prepared at desired concentrations after being filtered through a 0.22 μm filter. Samples were incubated overnight at room temperature with gentle rotation to allow fibril formation. TEM specimens were prepared by adding 8 μl of fibril solution to glow discharged carbon-coated copper grids for 1 min. The grids were then washed with distilled water and negatively stained with 2% uranyl acetate for 20 s followed by another wash. Grids were dried with filter paper wicking off excessive solution between each washing step. Samples were imaged using a Tecnai G2TF30 microscope (FEI Company, Eindhoven, The Netherlands) operating at 100 kV.

### X-ray fibril diffraction

RdlB fibrils were prepared in 10 mM sodium phosphate buffer pH 7.0 at a peptide concentration of 100 μM and left overnight at room temperature on a rocking platform shaker set at low speed (Ratek Instruments, VIC, Australia). Fibrils were pelleted by centrifugation at 1500 × *g* for 10 min and then resuspended in distilled water. Buffer salts were removed by repeating the centrifugation and resuspension steps three times. The pelleted RdlB fibrils were then resuspended in a small volume of distilled water at a final concentration of 2 mM. The dry stalk samples were prepared by suspending aliquots of fibril-containing solutions (8–10 μl) between two wax-filled capillary ends and allowed to dry in air at room temperature, as previously described^50^.

X-ray diffraction images were collected at the Australian Synchrotron on the macromolecular crystallography beamline (MX2) at a wavelength of 0.954 Å^51^. The sample to detector distance was 300 mm and the exposure time was 5 s. The 2-D diffraction images were converted into jpeg files using the program ADXV^52^. The diffraction patterns were radially integrated in both axial and equatorial directions on either side of the reflection within a 50° sector in the azimuthal direction using the analysis program Clearer^53^. The positions of the reflections and associated errors were automatically calculated by Clearer based on the positions of the maximal intensity on either side of the diffraction pattern. The average radial intensity was plotted against reciprocal spacing (1/d Å^−1^) for both the axial and equatorial direction.

### Scanning Electron Microscopy

Cryo-SEM was performed on a JEOL JSM-6700F. Six day old colonies of *S. coelicolor* were cut from solid MS agar medium and transferred onto the surface of an aluminum stub and frozen in nitrogen slush. Samples were subsequently transferred to the cryo-stage of the microscope. After the surface frost was sublimated (10 min at −90 °C), samples were coated with platinum plasma for 3–5 minutes at 7 mA at −120 °C. Samples were then moved onto the cryostage held at −120 °C and viewed at 3.0 kV.

## Additional Information

**How to cite this article**: Yang, W. *et al*. The propensity of the bacterial rodlin protein RdlB to form amyloid fibrils determines its function in *Streptomyces coelicolor. Sci. Rep.* **7**, 42867; doi: 10.1038/srep42867 (2017).

**Publisher's note:** Springer Nature remains neutral with regard to jurisdictional claims in published maps and institutional affiliations.

## Supplementary Material

Supplementary Information
