# Supplementary material for: The propensity of the bacterial rodlin protein RdlB to form amyloid fibrils determines its function in Streptomyces coelicolor
Source: Sci Rep. 2017 Feb 17;7:42867. doi: 10.1038/srep42867 (PMC5314377; doi:10.1038/srep42867)
Supplement: Supplementary Information [file srep42867-s1.pdf]

SUPPLEMENTARY INFORMATION

The propensity of the bacterial rodlin protein RdlB to form amyloid fibrils determines its function in *Streptomyces coelicolor*

Wen Yang[1,#], Joost Willemse[2], Elizabeth B. Sawyer[1,¶], Fei Lou[1,4], Weibin Gong[1], Hong Zhang[1,4], Sally L. Gras[3,*], Dennis Claessen[2,*] and Sarah Perrett[1,4*]

[1]National Laboratory of Biomacromolecules, CAS Center for Excellence in Biomacromolecules, Institute of Biophysics, Chinese Academy of Sciences, Beijing, 100101, China.

[2]Molecular Biotechnology, Institute of Biology, Leiden University, 2333 BE Leiden, the Netherlands.

[3]Department of Chemical and Biomolecular Engineering and Bio21 Molecular Science and Biotechnology Institute, The University of Melbourne, Parkville, 3010 VIC, Australia.

[4]University of the Chinese Academy of Sciences, Beijing 100049, China.

*Correspondence: sgras@unimelb.edu.au, d.claessen@biology.leidenuniv.nl, sarah.perrett@cantab.net

[#]Current address: Leiden University, 2333 BE Leiden, the Netherlands.

[¶]Current address: London School of Hygiene and Tropical Medicine, London WC1E 7HT, UK.

Table S1. Strains and plasmids used in this study

	Description	Reference
**Streptomyces**		
M145	*S. coelicolor* wild-type strain	1
ΔrdlB	*S. coelicolor* rdlB knocked-out strain	2
ΔrdlAB	*S. coelicolor* rdlA and rdlB double knocked-out strain	2
**Plasmids**		
pIJ8630	*E. coli-Streptomyces* shuttle vector	1
pHP45Ωhyg	Plasmid containing the hygromycin resistance fragment	3
pBluescript-II KS+	*E. coli* cloning vector	Stratagene
pBluescript-rdlB	pBluescript-II containing a 1.4 kb fragment encompassing the putative promoter and coding sequence of rdlB flanked with EcoRV	2
pBluescript-rdlA	pBluescript-II containing a 1.5 kb fragment encompassing the putative promoter and coding sequence of rdlA flanked with EcoRV	2
pBluescript-rdlB-	pBluescript-II containing a 1.4 kb rdlB- fragment flanked with EcoRV	This work
pBluescript-Δ17-42rdlB	pBluescript-II containing a 1.3 kb mutant Δ17-42rdlB fragment flanked with EcoRV	This work
pBluescript-rdlA*2	pBluescript-II carrying two 1.5 kb RdlA* fragment flanked with EcoRV and EcoRI separately	This work
pBluescript-rdlArdlB	pBluescript-II carrying a 1.5 kb RdlA fragment flanked with EcoRV and a 1.4 kb RdlB fragment flanked with EcoRI	This work
pIJ8630-rdlB-	pIJ8630 containing the 1.4 kb fragment encompassing the putative promoter and coding sequencing of RdlB- as well as the 2.2 kb hygromycin resistance fragment.	This work
pIJ8630-Δ17-42rdlB	pIJ8630 containing the 1.3 kb fragment encompassing the putative promoter and coding sequencing of mutant Δ17-42RdlB as well as the 2.2kb hygromycin resistance fragment.	This work
pIJ8630-rdlA*2	pIJ8630 containing the 3.0 kb fragment encompassing two copies of the putative promoter and the coding sequence of the triple mutant RdlA* as well as the 2.2kb hygromycin resistance fragment.	This work
pIJ8630-rdlArdlB	pIJ8630 containing one 1.5kb fragment encompassing the putative promoter and the coding sequence of RdlA and one 1.4 kb fragment encompassing the putative promoter and the coding sequence of RdlB, as well as the 2.2kb hygromycin resistance fragment.	This work

Table S2. Oligonucleotide primers used in this study for mutant construction

Name	Sequence(5'-3')	Use
F-Ra	CGCGGATCCATCGGGGACGACAACGGGCCGGCCGTGG	cloning RdlA
Nrev	CCGCAAGCTTTCGAGCTTAGCGGCCCTCG	cloning RdlA
F-Rb	CGCGGATCCATCGGCGACGACAGCGGGCCCGTCTCCG	cloning RdlB
RbR	GGGAAGCTTTCAGCCCTTGCCGCCCTCG	cloning RdlB
DB42R	GGGAAGCTTTCACGGCTTGTTGAACGAGCCC	1-42RdlB
DB43F	CGCGGATCCTGCATCGCGGTCAGCGACATCCC	43-105RdlB
B31-42F	CATGAGCCCGTGCATCGCGGTCAGCGACATCC	Δ31-42RdlB
B31-42R	CGCGATGCACGGGCTCATGTTGCCCGTGG	Δ31-42RdlB
1729 Fwd	ACGGCGCCTCGCCGCAGATGGCGCTCATCCAGGG	Δ17-29RdlB
1729 Rev	ATCTGCGGCGAGGCGCCGTTCCCGTTGGC	Δ17-29RdlB
17-42 Fwd	CGGCGCCTCGTGCATCGCGGTCAGCGACATCCCGG	Δ17-42RdlB
17-42 Rev	GACCGCGATGCACGAGGCGCCGTTCCCGTTGGCGG	Δ17-42RdlB
D51-57F	GACATCCCGATCCAGGACCTCAACGTCC	Δ51-57RdlB
D51-57R	GTCCTGGATCGGGATGTCGCTGACCGCGATGC	Δ51-57RdlB
RevT1:	AGTACTCCGAGGCGCCGTTCCCG	RdlB– mutant
ChainF	GCCTCGGAGTACTTCGGCAACTCGATGACCACGGGCGAC ATGAGCCCGCAGATGGCGCTCATCGAGGGCTCGT	RdlB– mutant
ChainR	ACGAGCCCTCGATGAGCGCCATCTGCGGGCTCATGTCGC CCGTGGTCATCGAGTTGCCGAAGTACTCCGAGGC	RdlB– mutant
FwdT3	ATCGAGGGCTCGTTCAACAAGC	RdlB– mutant
Rev T2:	ACGAGCCCTCGATGAGCGCCATCTGC	RdlB– mutant
Fwd T2	CGCCTCGGAGTACTTCGGCAACTCG	RdlB– mutant
B-1F	TACTTCGGCGACTCGATGACCACGGG	RdlB– mutant
B-1R	TCATCGAGTCGCCGAAGTACTCCGAGGC	RdlB– mutant
B-2F	ATGAGCCCGGAGATGGCGCTCATCG	RdlB– mutant
B-2R	AGCGCCATCTCCGGGCTCATGTCGCCCG	RdlB– mutant
B-3F	AGGGCTCGTTCGACAAGCCGTGCATCGC	RdlB– mutant
B-3R	ACGGCTTGTCGAACGAGCCCTCGATGAGC	RdlB– mutant
A1F	AACGGCGCCCAGTCGGCGTTCGGCAACTCGG	RdlA* mutant
A1R	AACGCCGACTGGGCGCCGTTGCCGTTGGC	RdlA* mutant
A2F	CACCAAGGGCAACATGAGCCCCCAGCTGTCG	RdlA* mutant
A2R	GGGCTCATGTTGCCCTTGGTGGCCGAGTTGC	RdlA* mutant
A3F	TCGCTGGTCCAGGGCACGCTGAACAAGC	RdlA* mutant
A3R	AGCGTGCCCTGGACCAGCGACAGCTGGG	RdlA* mutant
DA-F	CGGCCGGAATTCGATGATATCATCAAGCTTATCG	pBluescript-rdlA*2
DA-R	GCCGGAATTCTGGCCAGTCCTTTCTGAGG	pBluescript-rdlA*2
DB-F	GCCGGAATTCTTCGCGTTCCTTTCGTCGTAGC	pBluescript-rdlArdlB
DB-R	GCCGGAATTCGCCACCATCGTCCCGC	pBluescript-rdlArdlB

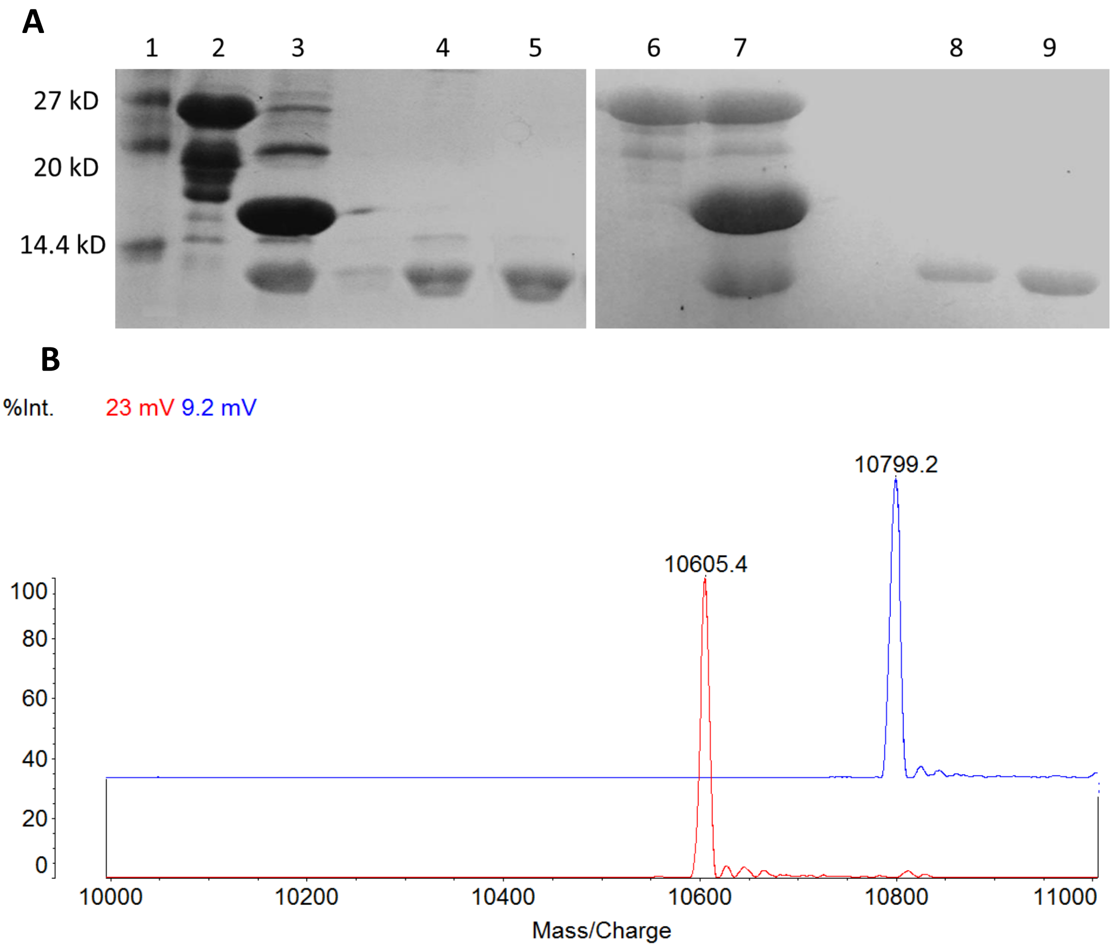

Figure S1. SDS-PAGE and MALDI-TOF mass spectrometry of recombinant RdlA and RdlB produced in *E. coli* and purified by Ni affinity chromatography and size exclusion chromatography. (**A**) SDS-PAGE. Lane 1: Marker; Lane 2: His6-Smt3-RdlA after the first Ni affinity chromatography purification step; Lane 3: His6-Smt3 and RdlA (the lowest band) produced from Ulp1 cleaved His6-Smt3-RdlA; Lane 4: RdlA after the second Ni affinity chromatography step; Lane 5: RdlA after size exclusion chromatography; Lane 6: His6-Smt3-RdlB after the first Ni affinity chromatography purification step; Lane 7: His6-Smt3 and RdlB (the lowest band) produced from Ulp1 cleaved His6-Smt3-RdlB; Lane 8: RdlB after the second Ni affinity chromatography purification; Lane 9: RdlB after size exclusion chromatography. (**B**) MALDI-TOF mass spectrometry of purified RdlA and RdlB. Peaks corresponding to RdlA and RdlB are labeled along with their detected mass/charge ratios. Note that an extra serine residue remains at the N-terminus of the rodlin proteins as a result of ULP1 cleavage of the SMT3 fusion tag. The theoretical molecular mass values for RdlA and RdlB, including the additional serine residue, are 10603 Da and 10796 Da, respectively.

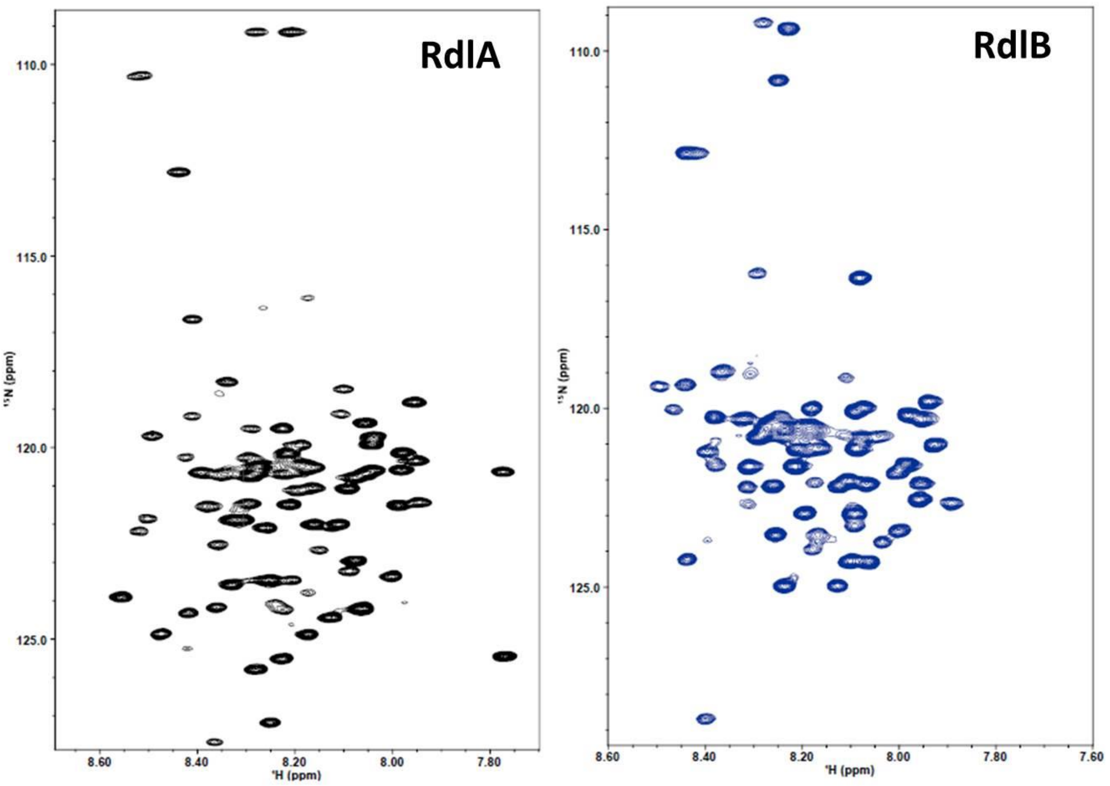

Figure S2. NMR characterization of the recombinantly expressed RdlA and RdlB without the 28 residue putative signal peptide at the N-terminus. The ^{1}H-^{15}N HSQC spectra of RdlA (*left*) and RdlB (*right*) suggest that both proteins are intrinsically disordered, as the backbone NH signals of the two proteins are located in a very narrow region less than 1.0 ppm wide, spanning 7.7-8.6 ppm for RdlA and 7.8-8.5 ppm for RdlB.

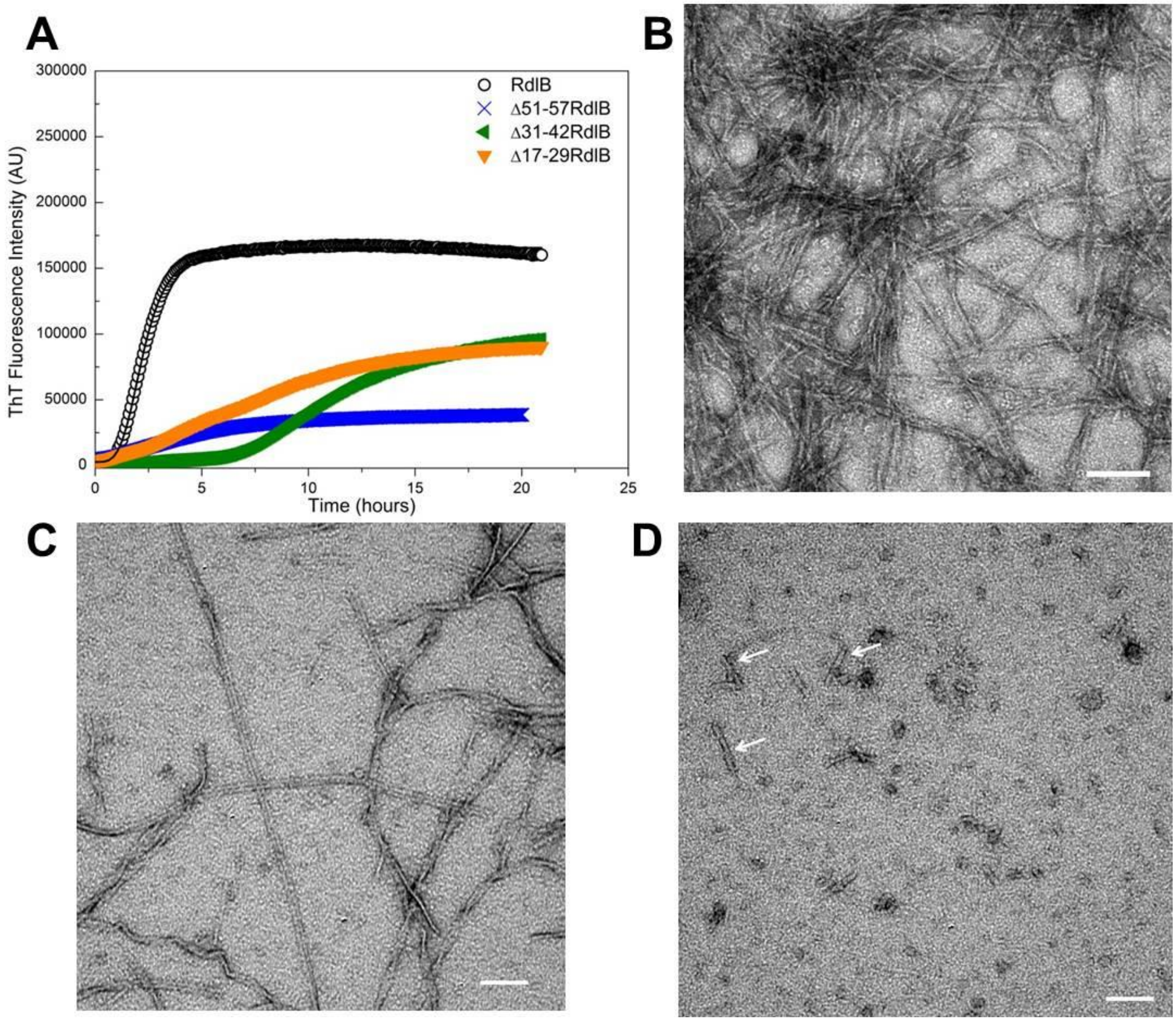

Figure S3. Three deletion mutants of RdlB show different propensities to form amyloid fibrils *in vitro* compared to wild type RdlB. (**A**) Aggregation of Δ51-57RdlB, Δ31-42RdlB, Δ17-29RdlB and full length RdlB monitored by ThT fluorescence. All four proteins were at a concentration of 10 μM. Readings were taken every 3 mins for over 20 hours in a microplate reader with 700 rpm orbital shaking. (**B**) Mutant Δ51-57RdlB efficiently self-assembles into amyloid fibrils. Abundant mature Δ51-57RdlB fibrils were observed by TEM after incubation at room temperature for 24 hours although the fluorescence of ThT in the presence of these fibrils is lower. (**C**) Mutant Δ31-42RdlB is still capable of forming fibrils even though the ThT fluoresencence data suggests a significantly extended lag time. (**D**) Fibril formation by mutant Δ17-29RdlB seems severely impeded, as only a few short fibrils are occasionally found by TEM although the increase in the fluorescence of the ThT in the presence of these fibrils appears relatively high. These short fibrils are indicated by arrows. (**B-D**) The scale bars are 100 nm in length.

A

```
                10        20        30        40
RdlA   IGDDNGPAVANGNGAESAFGNSATKGDMSPQLSLVEGTLNKP
RdlB   IGDDSGPVSANGNGASQYFGNSMTTGNMSPQMALIQGSFNKP
       **** **  ******   **** * * ****  *  *  ***
RdlB*  IGDDSGPVSANGNGASEYFGNSMTTGDMSPQMALIEGSFNKP
```

B

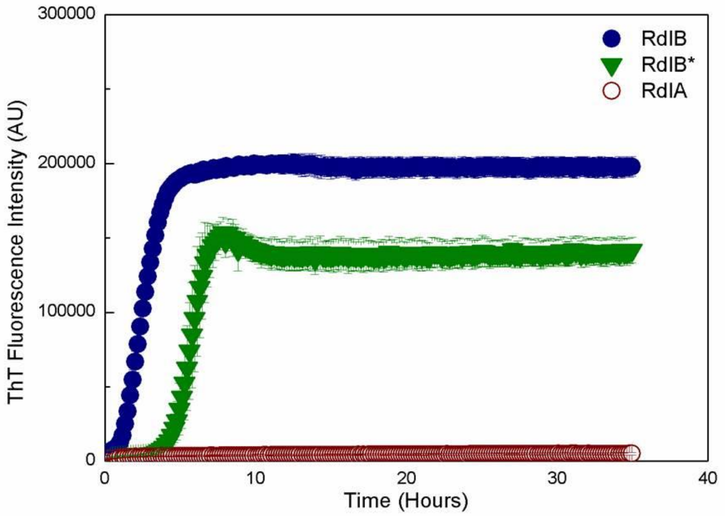

Figure S4. Triple site mutant RdlB* retains amyloid forming ability. (**A**) Sequence alignment of the N-terminal regions of rodlins and the mutant RdlB*. (**B**) The aggregation of wild-type rodlins and the mutant RdlB* monitored by ThT fluorescence. The data shown are the average of at least 4 replicates and the error bars represent the standard error of the mean.

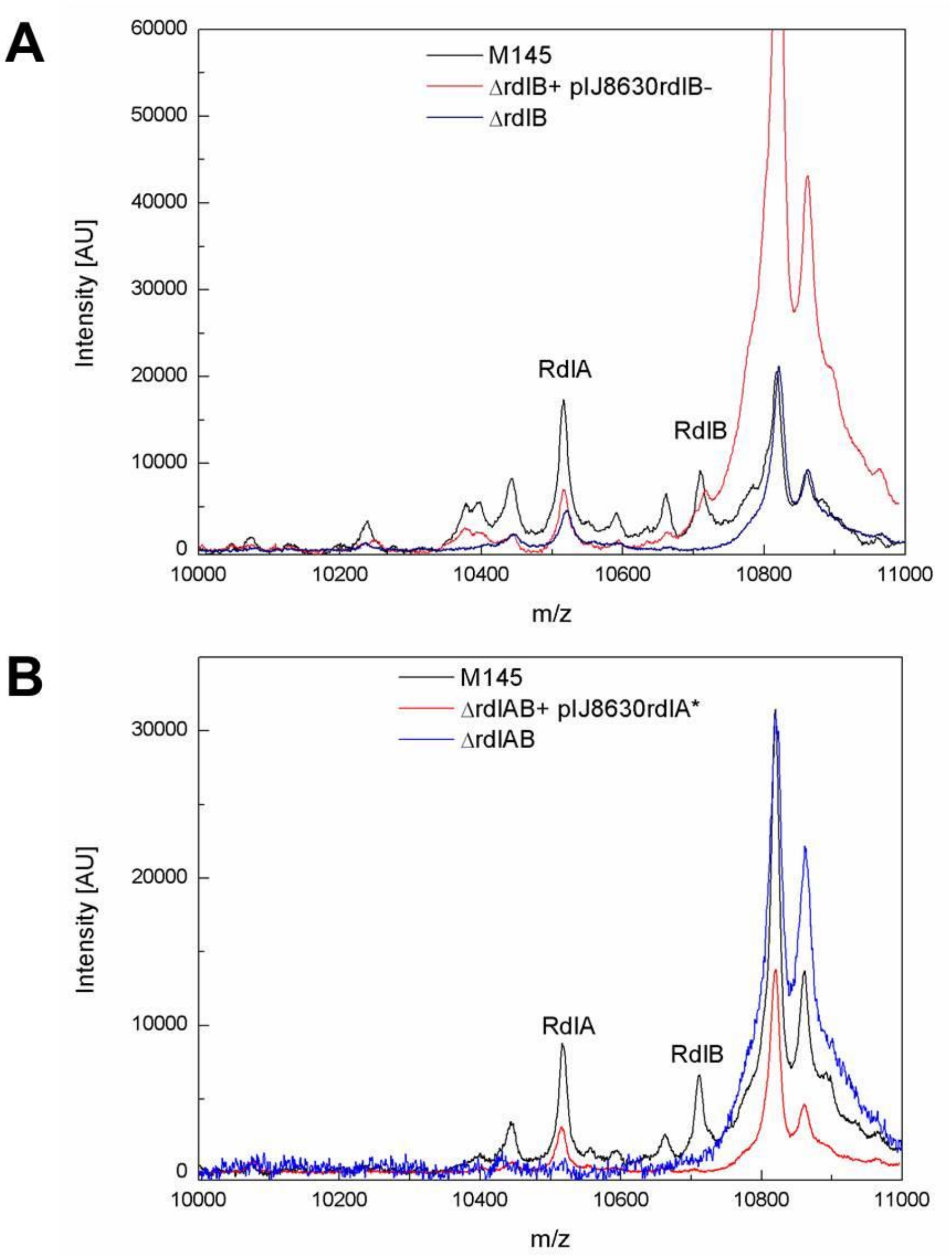

Figure S5. MALDI-TOF mass spectrometry analysis of spores of the *S. coelicolor* strain Δ*rdlB* complimented with pIJ8630-rdlB- and strain Δ*rdlAB* complimented with pIJ8630-rdlA*. Spores of different strains were picked from 6 day old colonies on solid MS agar medium with a toothpick and mixed with 2 µl matrix solution containing a-cyano-4-hydroxycinnamic acid (10 mg/mL in 50% acetonitrile/0.1% trifluoroacetic acid) spotted on plate[4]**.** Samples were analysed using a Microflex MALDI-TOF Mass Spectrometer (Bruker, Bremen, Germany). (**A**) In wild type M145 the two peaks of mass/charge at 10516.68 Da and 10711.09 Da can be identified as RdlA and RdlB, respectively. The strain Δ*rdlB* complimented with pIJ8630rdlB– shows two peaks at 10515.94 Da and 10715.70 Da representing the appearance of RdlA and RdlB–, respectively. Only the RdlA peak is identified in strain ΔrdlB. (**B**) In the strain ΔrdlAB complimented with pIJ8630-rdlA* the peak at 10516.07 Da corresponds to RdlA*, while there is no peak detected for RdlB, as expected. Both Rodlin peaks are missing in strain ΔrdlAB.

A

B

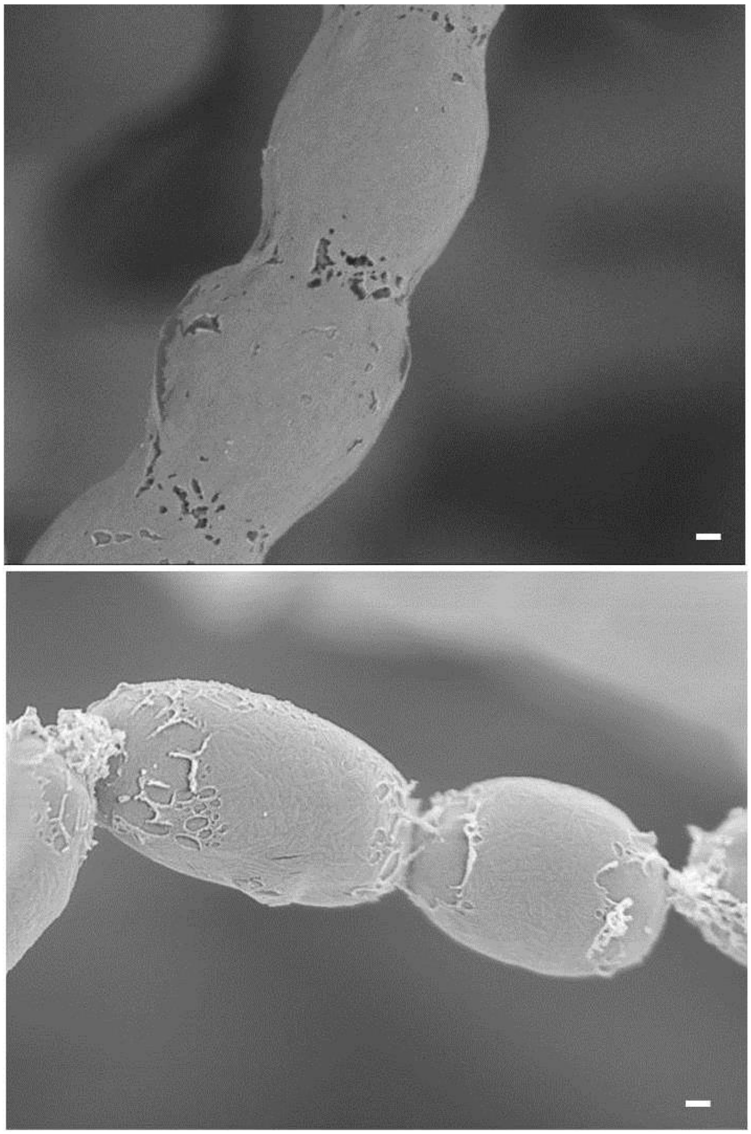

Figure S6. The spore surface does not exhibit the rodlet layer in the double knock-out strain Δ*rdlAB* (**A**). In the double knock-out strain Δ*rdlAB* complemented with plasmid pIJ8630-rdlArdlB the rodlet layer is restored (**B**). (**A,B**) The scale bar is 100 nm in length.

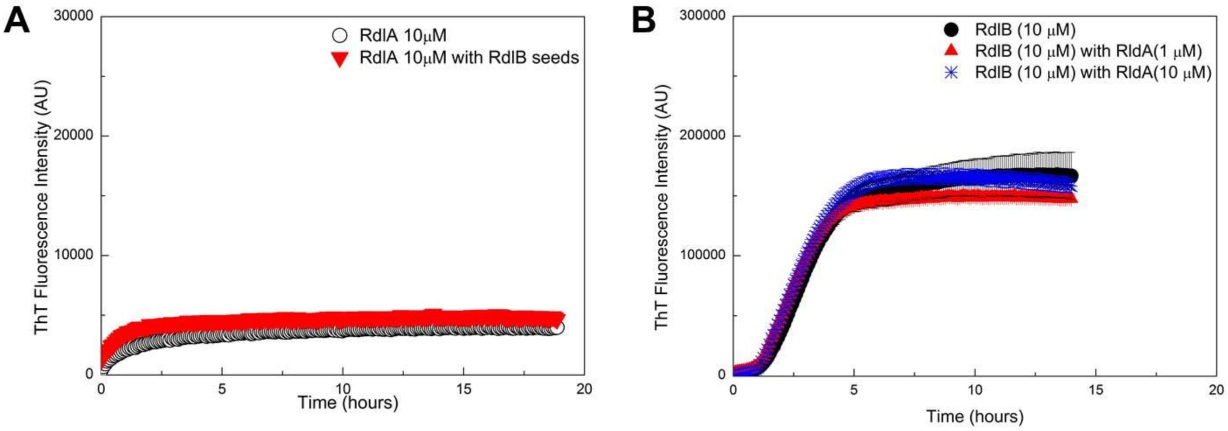

Figure S7. ThT assay suggests no seeding effect between RdlA and RdlB. (**A**) RdlA aggregation monitored by ThT fluorescence intensity with or without RdlB fibrils seeds at 10% of the concentration of RdlA (10μM). (**B**) RdlB (10μM) aggregation monitored by ThT fluorescence intensity in the presence or absence of different concentrations of RdlA, as indicated. (**A,B**) Readings were taken every 3 min in a FLUOstar Omega microplate reader with 700 rpm orbital shaking. The data shown are the average of at least 4 replicates and the error bars represent the standard error of the mean. Note that the y-axis representing the fluorescence intensity in panel **A** is 10 times smaller than in panel **B**.

References

1 Kieser, T., Bibb, M., Buttner, M., Chater, K. & Hopwood, D. *Practical Streptomyces genetics.* (The John Innes Foundation, 2000).

2 Claessen, D. *et al.* The formation of the rodlet layer of streptomycetes is the result of the interplay between rodlins and chaplins. *Mol. Microbiol.* **53**, 433-443 (2004).

3 Prentki, P. & Krisch, H. M. In vitro insertional mutagenesis with a selectable DNA fragment. *Gene* **29**, 303-313 (1984).

4 de Jong, W., Vijgenboom, E., Dijkhuizen, L., Wösten, H. A. B. & Claessen, D. SapB and the rodlins are required for development of *Streptomyces coelicolor* in high osmolarity media. *FEMS Microbiol. Lett.* **329**, 154-159 (2012).
